# Supplementary figures and images for: Novel aspects of iron homeostasis in pathogenic bloodstream form Trypanosoma brucei
Source: PLoS Pathog. 2021 Jun 23;17(6):e1009696. doi: 10.1371/journal.ppat.1009696 (PMC8259959; doi:10.1371/journal.ppat.1009696)

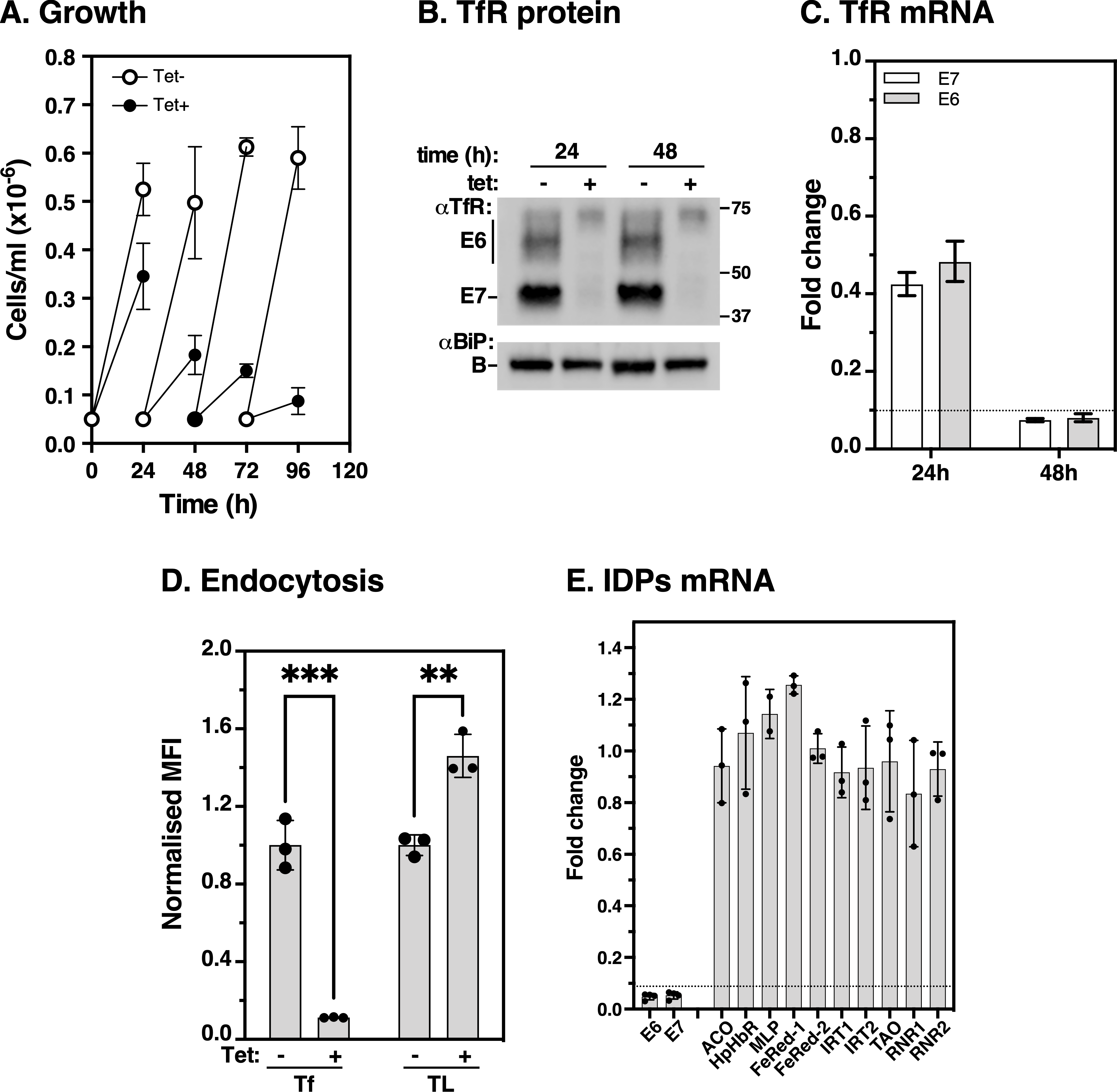

Supplement: S1 Fig — A tetracycline inducible RNAi cell line was generated targeting all subunits of native transferrin receptor E6 and E7. A. Growth curves of cells cultured in the presence (Tet+) or absence (Tet-) of 1 μg/ml tetracycline. Number of parasites/ml on the y-axis adjusted to starting density daily vs time on x-axis (days). Data are means ± SD (n = 3 technical replicates). B. TfR subunits were pulled down with holo-Tf conjugated beads from RNAi un-induced (tet -) or induced (tet +) cell lysates and detected by blotting with either anti-TfR (αTfR) at time points indicated. Anti-BiP (αBiP) serves as loading control. C. Relative TfR mRNA expression was analysed by qRT-PCR at indicated time points post RNAi inductions, normalized to un-induced cells at each time point. Data are means ± SD (n = 3 technical replicates, with 3 technical replicates for each n). D. Live cells were treated with tetracycline (Tet+) to initiate specific dsRNA synthesis or untreated (Tet-) and uptake of fluorescent-labelled holo-transferrin (Tf) or tomato lectin (TL) was analysed by flow cytometry. Normalised median fluorescent intensity (MFI) are shown for three independent biological replicates relative to un-induced controls. TL uptake serves as a surrogate control for general endocytosis. Data are means ± SD (n = 3 biological replicates). ***P < 0.001, **P < 0.002, as determined by Student t-test. E. qRT-PCR was performed to evaluate the effect of iron starvation following TfR RNAi on known homologues of iron-dependent proteins (IDPs). Relative mRNA levels of selected homologues of IDPs in BSF trypanosomes are shown. The relative quantity of each transcript was normalized against ZFP3 (Tb927.3.720) relative to un-induced controls. Abbreviations: E6 (ESAG6), E7 (ESAG7), RBP5 (RNA-binding Protein 5; Tb927.11.12100), PAP2 (Phosphatidic Acid Phosphatase; Tb927.8.480), Hyp#1 (hypothetical protein; Tb927.8.490), Hyp#2 (hypothetical protein, Tb927.8.510); FeRed-1 (Ferric Reductase, Tb927.6.3320), FeRed-2 ( [file ppat.1009696.s001.tif]

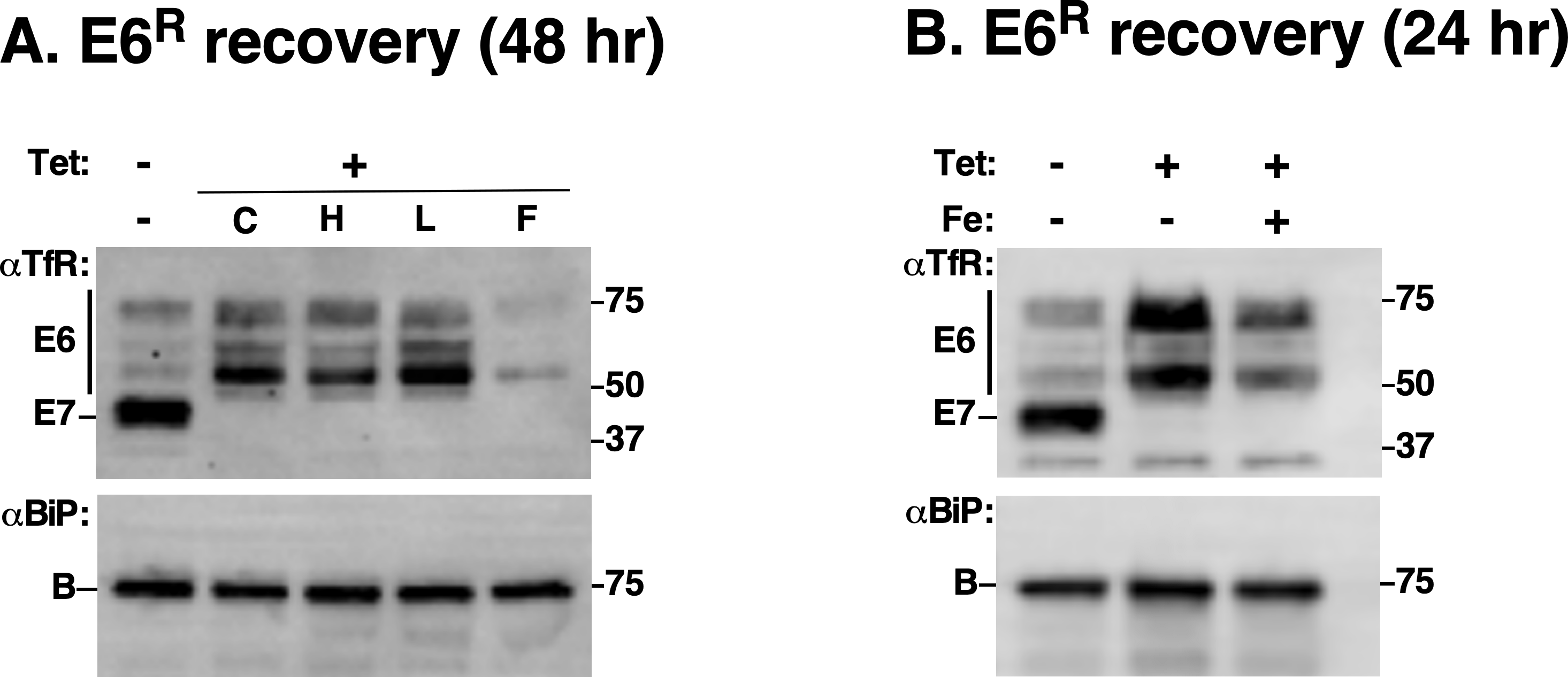

Supplement: S2 Fig — A. E6R expressing cells were cultured in the presence of 1 μg/ml tetracycline (Tet +) supplemented with holo-transferrin (H, 200 μg/ml), Lactoferrin (L, 200 μg/ml), FeCl3 (F, 25 μM) or untreated (C) for 48 hr and total protein was extracted and blotted with αTfR or αBiP (loading control). B. E6R expressing cells were pretreated with tetracycline for 24 hr followed by supplementation with FeCl3 for an additional 24 hr, and total protein blotted as in (A). (TIF) [file ppat.1009696.s002.tif]

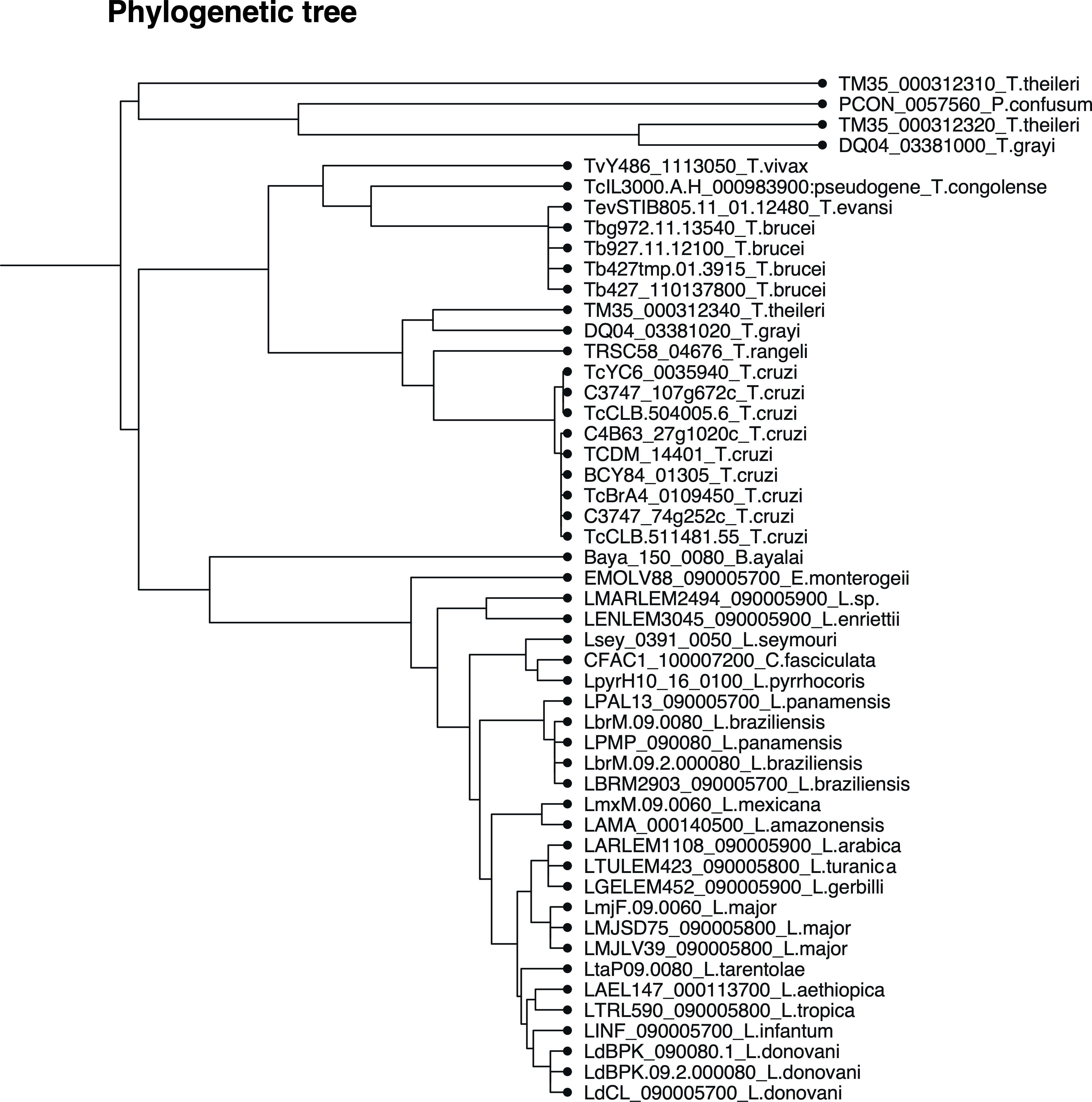

Supplement: S3 Fig — Proteins with homology to RBP5 were retrieved from TritrypDB and the output was saved as public search: https://tritrypdb.org/tritrypdb/app/workspace/strategies/import/3ef3dfbc6f322bf6. The sequences were imported in Jalview and aligned with the Mafft alghoritm (L-INS-i) using the Jalview interface. An aligned region of 102 amino acids with no gaps was used to create a phylogenetic tree with the Jalview interface (Average Distance and BLOSUM62 matrix). The tree was exported in newick format and loaded in FigTree. Proteins with accession numbers: TM35_000312320 and TM35_000312310 (Trypanosoma theileri), PCON_0057560 (Paratrypanosoma confusum) and DQ04_03381000 (Trypanosoma grayi) cluster together but very divergent relative to the other putative RBP5 proteins. For this reason, the alignment was rerouted using these divergent sequences as outgroup. (TIF) [file ppat.1009696.s003.tif]

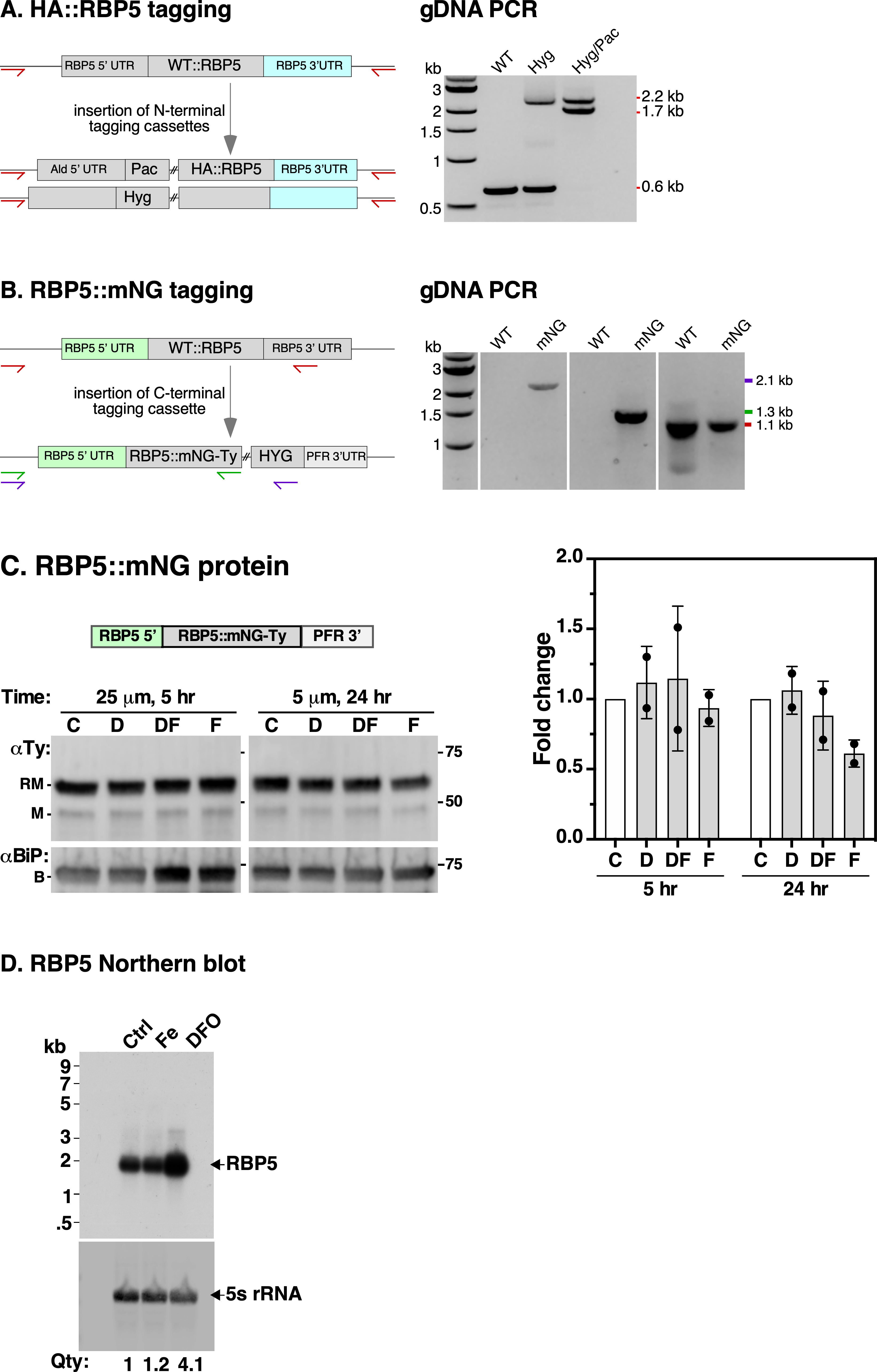

Supplement: S4 Fig — A. In situ N-terminal tagging of RBP5 with 6xHA retaining its native 3’-UTR. Selection of transgenic cells was achieved using puromycin (PAC) and Hygromycin (Hyg). Arrows indicate position of PCR primers to verify RBP5 locus-specific integration. The corresponding size fragments are shown by genomic DNA (gDNA) PCR from wild type (WT), single allele (Hyg) or both alleles (Hyg/Pac) tagged cell lines. All diagrams are not drawn to scale and in situ tags were done by CRISPR/Cas9-mediated PCR-based method published in [36]. B. Schematic shows strategy for generating in situ C-terminally tagged RBP5 (RBP5::mNG-Ty) fused with PFR 3’-UTR and hygromycin (HYG) as drug selection cassette. Arrows indicate position of PCR primers to analyse correct integration with corresponding size fragments shown in agarose gel on the right. CRISPR/Cas9 mediated tagging strategy in (a) was used. C. Effect of iron starvation on C terminal epitope tagged RBP5 protein expression. Schematic of C-terminal in situ tagged RBP5 (RBP5::mNG-Ty) fused with PFR 3’-UTR. RBP5::mNG-Ty expressing cells were incubated with deferoxamine [25 μM, 5 hr or 5 μM, 24 hr]. Total protein was extracted and blotted with αTy or αBiP antibodies. RM, M indicate RBP5::mNG-Ty and mNG::Ty polypeptides, respectively. Bar chart (right) shows quantification of RBP5::mNG-Ty relative to BiP signals. D. Effect of iron starvation on RBP5 mRNA abundance by Northern blot analyses. Log phase BSF cells (~5x10^5^ cells/ml) were incubated with deferoxamine (DFO), Iron (III) chloride (Fe) or untreated (Ctrl), RNA was isolated and analysed by Northern blotting with probes to full length RBP5 ORF plus 1,235 bp downstream of the STOP codon. The blot was re-probed with 5.8S rRNA as loading control [70]. For each sample, RBP5 mRNA levels were quantified relative to 5.8s rRNA and shown as Qty beneath the blot. In contrast to curated and experimental data of poly A sites from TritrypDB, these data (from a single experiment) show that the RBP5 3’-UT [file ppat.1009696.s004.tif]

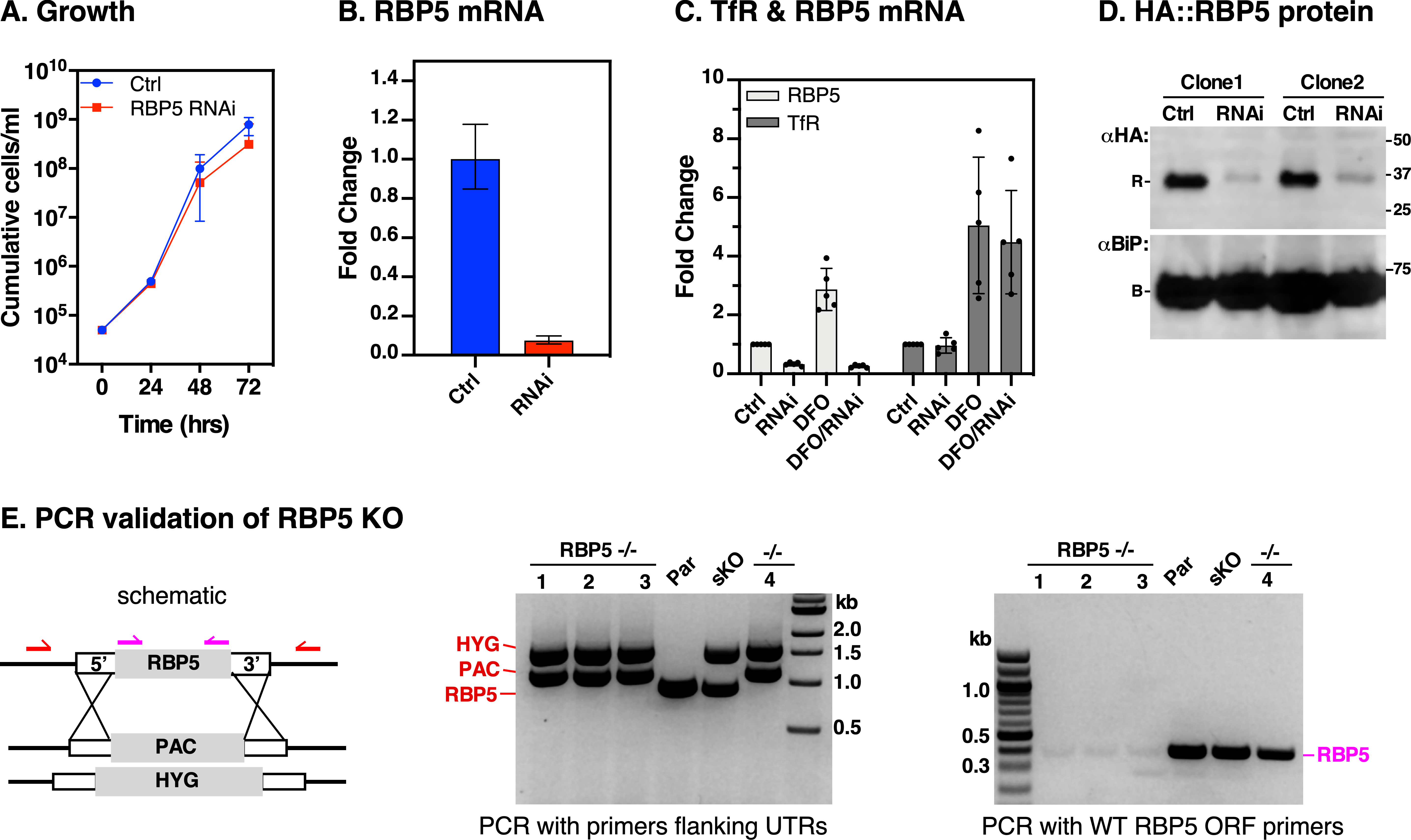

Supplement: S5 Fig — A Effect of RBP5 RNAi on cell growth. RBP5 RNAi cells were counted using a haemocytometer for 72 hrs with (RBP5 RNAi) or without (Ctrl) 1 μg/ml tetracycline. The value on the y-axis represents measured value times the dilution factor. Data are means ± SD, n = 3. B. qRT-PCR analyses showing levels of RBP5 mRNA with or without RBP5 RNAi at 48 hr, 1 μg/ml tetracycline. Data are means ± SD, n = 3 technical replicates. C. qRT-PCR analyses showing levels of RBP5 and TfR mRNA following treatment with 1 μg/ml tetracycline for 25 hr (RNAi), followed by 25 μM DFO for 5 hr (DFO), combined DFO and tetracycline (DFO/RNAi) or no treatment (Ctrl). Data means ± SD, n = 5 biological replicates, with 3 technical replicates for each n. D. Effect of RBP5 RNAi on HA::RBP5 protein levels. RBP5 RNAi construct was transfected into HA::RBP5 expressing cells, RNAi was induced for 24 hr (RNAi) or not (Ctrl) and protein levels determined by western blots with either anti-HA (R, RBP5) or anti-BiP (B, loading control) antibodies. E. Generation and PCR validation of RBP5 knockout cell lines. The schematic shows the targeting strategy of the RBP5 wild type locus. The arrows indicate the position of oligonucleotide primers used for PCR confirmation of the inserted drug resistance cassettes: Hygromycin (HYG) and Puromycin (PAC). The agarose gel images on the right show PCR amplicons obtained using genomic DNA template derived from either wild type (Par), RBP5 single knockout with HYG (sKO), or RBP5 double knockout (RBP5-/-) with HYG and PAC cassettes. The colours indicate the primers used as shown on the schematic. Four RBP5-/- clonal cell lines were tested from two independent transfections. In all instances, integration of both HYG and PAC cassettes were confirmed by PCR. However, the RBP5 ORF had been duplicated in the genome as confirmed by a PCR product using primers that anneal to the ORF. (TIF) [file ppat.1009696.s005.tif]

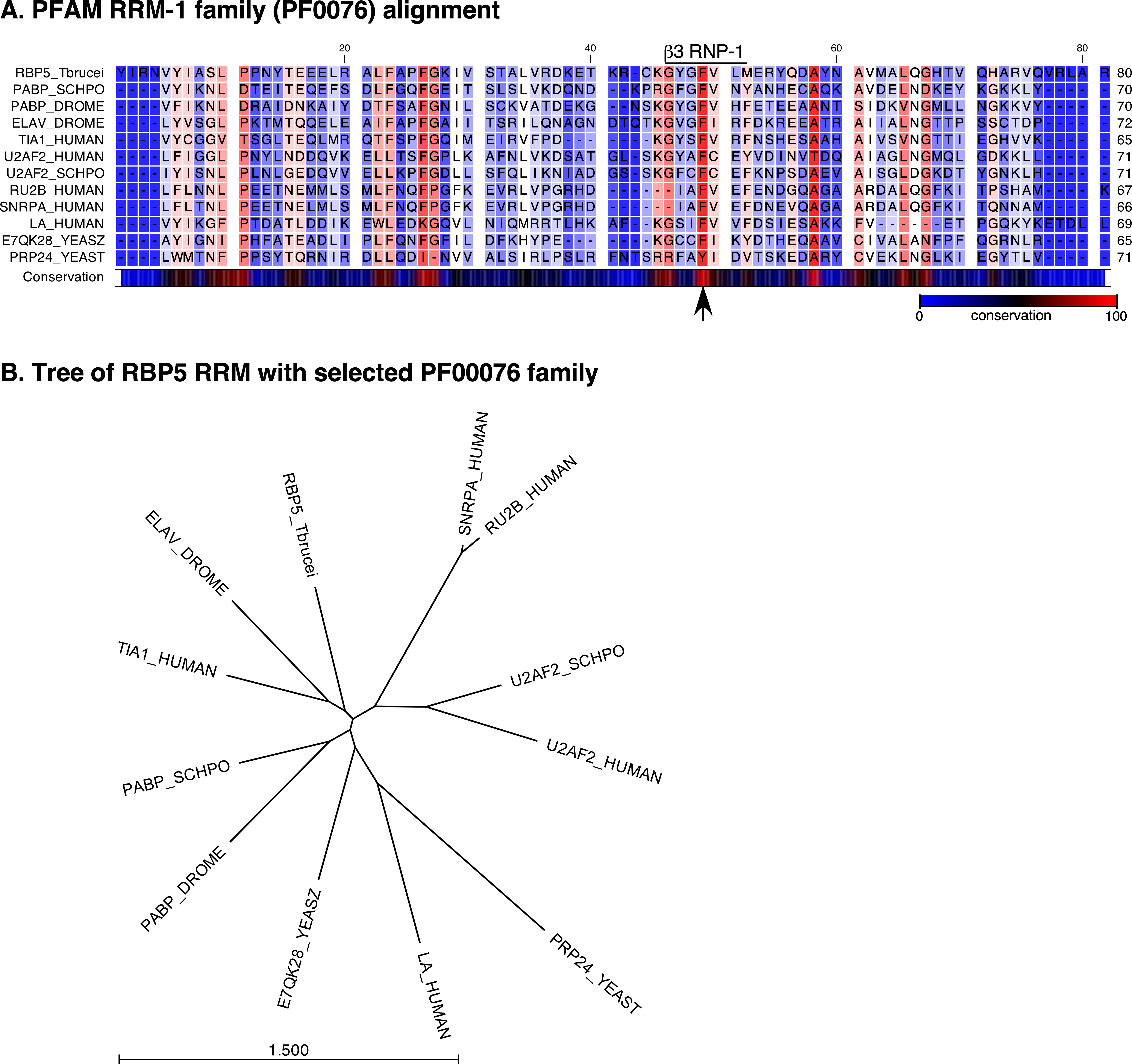

Supplement: S6 Fig — A. Selected RRM-1 Family (PF0076) proteins from different species were aligned with the RRM domain of T. brucei RBP5. Arrowhead shows conserved Phe residue RNP-1 motif. The residue was mutated to Ala based on conservation analysis using Jensen-Shannon divergence, structural and mutation analyses by SuSPect and Missense 3D. Mutation to alanine maintains hydrophobicity at this position potentially disrupting binding to target RNA sequence [54]. Abbreviations: PABP: poly(A) binding protein; SCHOP: Schizosaccharomyces pombe: DROME: Drosophila melanogaster; ELAV: Embryonic Lethal Abnormal Vision; TIA: T-cell-restricted intracellular antigen-1; U2AF: general splicing factor; U2AF2: U2 Small Nuclear RNA Auxiliary Factor 2; RU2B: small nuclear ribonucleoprotein polypeptide B2; SNRPA: U1 small nuclear ribonucleoprotein A; PRP24: Precursor RNA processing, gene 24. B. Radial tree of multiple sequence alignment in (a). The tree was generated using CLC Genomics Workbench 20 with default parameters. (TIF) [file ppat.1009696.s006.tif]

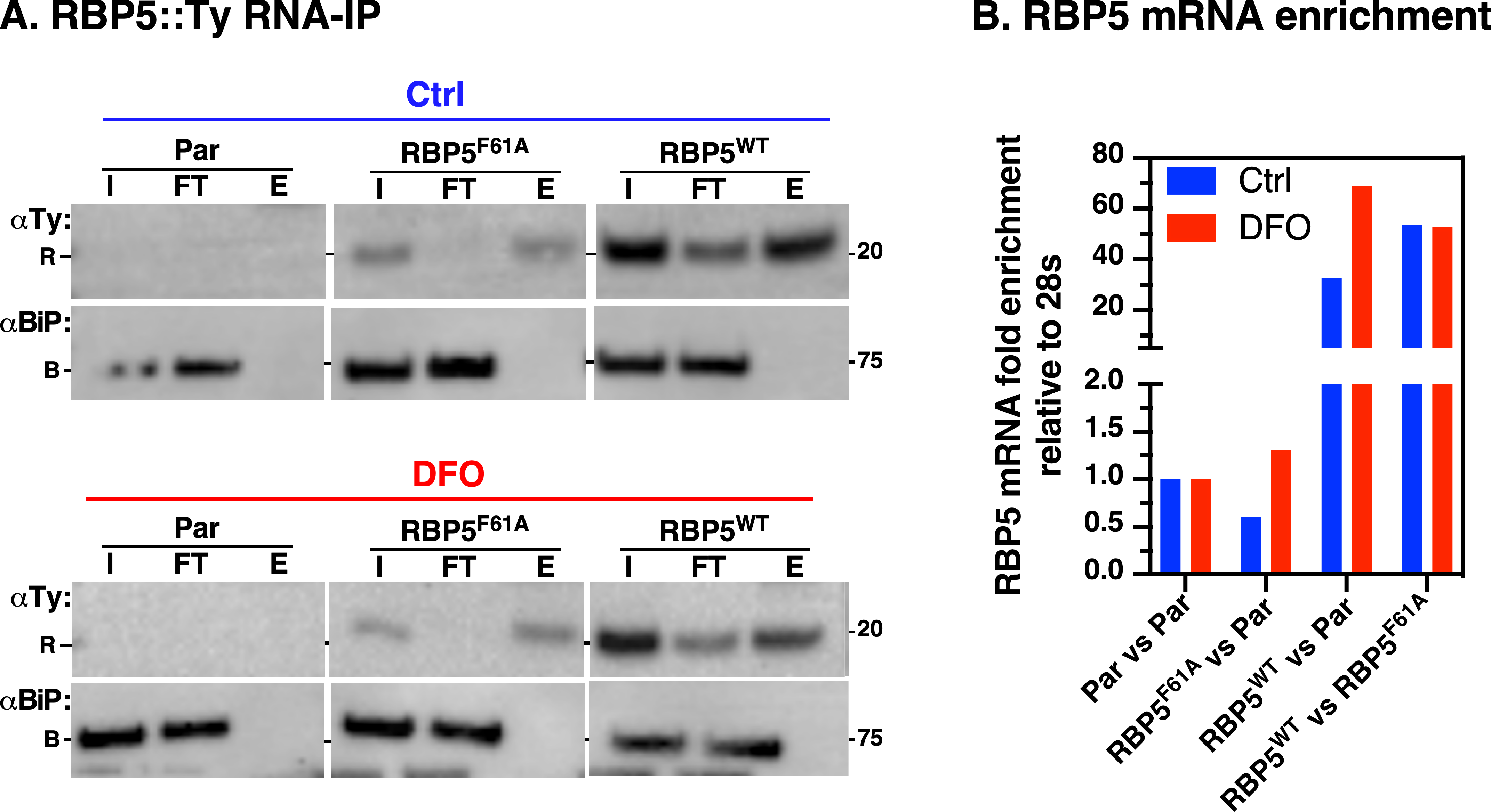

Supplement: S7 Fig — A. BSF cells expressing c-terminally Ty-tagged wild type RBP5 (RBP5WT), an RRM mutant (RBP5F61A) or untagged Parental (Par) strain were treated with tetracycline, supplemented with deferoxamine (DFO, bottom) or without (Control, top) for 4 hours. RIP was performed with anti-Ty beads, followed by immunoblotting with anti-Ty (αTy, R) and anti-BiP (αBiP, B). B. Bar chart shows relative fold-enrichment of RBP5 mRNA levels quantified by qRT-PCR and corrected for background either with untagged Par (RBP5WT vs Par) or with RBP5F61A (RBP5WT vs RBP5F61A). The value shown on the y-axis is normalised to 28s rRNA as non-bound control. Note that: (i) there was no enrichment of RBP5 mRNA in both untagged Par and RBP5F61A in either condition, as expected, (ii) bearing in mind that RBP5 protein is overexpressed, we see a 20–40 -fold RBP5 mRNA enrichment in Ctrl cells (blue bars). The data suggest that increased RBP5 protein expression is sufficient to elicit RBP5 protein/mRNA association. No conclusion can be made whether iron depletion by DFO in the context of overexpression enhances the interaction between RBP5 mRNA and protein since these data are derived from a single experiment. However, the data in Fig 8 showing endogenous HA-tagged protein levels reflects a physiological scenario. (TIF) [file ppat.1009696.s007.tif]
